# Radiomics Nomogram for Predicting Stroke Recurrence in Symptomatic Intracranial Atherosclerotic Stenosis

**DOI:** 10.3389/fnins.2022.851353

**Published:** 2022-04-12

**Authors:** Min Tang, Jie Gao, Niane Ma, Xuejiao Yan, Xin Zhang, Jun Hu, Zhizheng Zhuo, Xiaorui Shi, Ling Li, Xiaoyan Lei, Xiaoling Zhang

**Affiliations:** ^1^Department of Magnetic Resonance Imaging (MRI), Shaanxi Provincial People's Hospital, Xi'an, China; ^2^Department of Graduate, Xi'an Medical University, Xi'an, China; ^3^Department of Neurology, Shaanxi Provincial People's Hospital, Xi'an, China; ^4^Department of Radiology, Beijing Tiantan Hospital, Capital Medical University, Beijing, China

**Keywords:** intracranial arteriosclerosis, plaques, magnetic resonance imaging, stroke, recurrence, nomogram, radiomics

## Abstract

**Objective:**

To develop and validate a radiomics nomogram for predicting stroke recurrence in symptomatic intracranial atherosclerotic stenosis (SICAS).

**Methods:**

The data of 156 patients with SICAS were obtained from the hospital database. Those with and without stroke recurrence were identified. The 156 patients were separated into a training cohort (*n* = 110) and a validation cohort (*n* = 46). Baseline clinical data were collected from our medical records, and plaque radiological features were extracted from vascular wall high-resolution imaging (VW-HRMRI). The imaging sequences included 3D-T1WI-VISTA, T2WI, and 3D-T1WI-VISTA-enhanced imaging. Least absolute shrinkage and selection operator (LASSO) analysis were used to select the radiomics features associated with stroke recurrence. Then, multiple logistic regression analysis of clinical risk factors, radiological features, and radiomics signatures were performed, and a predictive nomogram was constructed to predict the probability of stroke recurrence in SICAS. The performance of the nomogram was evaluated.

**Results:**

Diabetes mellitus, plaque burden, and enhancement ratio were independent risk factors for stroke recurrence [odds ratio (OR) = 1.24, 95% confidence interval (CI): 1.04–3.79, *p* = 0.018; OR = 1.76, per 10% increase, 95% CI, 1.28–2.41, *p* < 0.001; and OR = 1.94, 95% CI: 1.27–3.09, *p* < 0.001]. Five features of 3D-T1WI-VISTA, six features of T2WI, and nine features of 3D-T1WI-VISTA-enhanced images were associated with stroke recurrence. The radiomics signature in 3D-T1WI-VISTA-enhanced images was superior to the radiomics signature of the other two sequences for predicting stroke recurrence in both the training cohort [area under the curve (AUC), 0.790, 95% CI: 0.669–0.894] and the validation cohort (AUC, 0.779, 95% CI: 0.620–0.853). The combination of clinical risk factors, radiological features, and radiomics signature had the best predictive value (AUC, 0.899, 95% CI: 0.844–0.936 in the training cohort; AUC, 0.803, 95% CI: 0.761–0.897 in the validation cohort). The C-index of the nomogram was 0.880 (95% CI: 0.805–0.934) and 0.817 (95% CI: 0.795–0.948), respectively, in the training and validation cohorts. The decision curve analysis further confirmed that the radiomics nomogram had good clinical applicability with a net benefit of 0.458.

**Conclusion:**

The radiomics features were helpful to predict stroke recurrence in patients with SICAS. The nomogram constructed by combining clinical high-risk factors, plaque radiological features, and radiomics features is a reliable tool for the individualized risk assessment of predicting the recurrence of SICAS stroke.

## Introduction

Intracranial atherosclerotic stenosis (ICAS) is a common cause of ischemic stroke, especially in Asian populations (Chimowitz et al., 2005; Wang et al., 2014). Stroke caused by ICAS is characterized by relatively severe symptoms, longer hospital stay, and a high risk of stroke recurrence (Wang et al., 2014). Even with active treatment, 12–38.2% of patients with ICAS will have a recurrent stroke within a year (Mazighi et al., 2006; Derdeyn et al., 2014; Prabhakaran et al., 2021). Effective secondary prevention can be facilitated by identification markers associated with recurrent stroke.

Stroke recurrence in ICAS is related to various factors, such as plaque burden, plaque inflammation, infarction pattern, collateral status, etc. (Kim et al., 2016; Wabnitz et al., 2019; Song et al., 2020). Plaque burden and plaque enhancement are independent risk factors for stroke recurrence, and a greater degree of stenosis is associated with a higher risk of recurrence (Ran et al., 2020; Shi et al., 2021). Vessel wall high-resolution magnetic resonance imaging (VW-HRMRI) can accurately detect plaque burden and also provide components, inflammation, and angiogenesis information (Saam et al., 2007), which are features that help the clinician assess plaque vulnerability and predict future cerebrovascular events (Underhill et al., 2010; Hosseini et al., 2013). Despite the great progress in high-resolution magnetic resonance imaging (HRMRI) evaluation of intracranial atherosclerotic plaques, limitations of imaging resolution and technical methods, complexity of intracranial arterial plaques, and subjectivity in the interpretation of plaque components and morphology make the accurate evaluation of intracranial atherosclerotic plaques challenging. There is an urgent need for automatic and reproducible quantitative methods to evaluate plaque characteristics predictive of recurrent stroke in symptomatic ICAS (SICAS).

Radiomics, which analyzes the high-throughput quantitative parameters in radiographic images, can obtain histological and biological information of whole lesions, has been shown to be useful for diagnosis and classification, and aids in the evaluation of treatment response of various tumors (Aerts et al., 2014; Yamamoto et al., 2014; Gillies et al., 2016; Kickingereder et al., 2016; Braman et al., 2017). Radiomics methods are gradually being applied for the diagnosis of intracranial atherosclerotic diseases and has been shown to be capable of distinguishing symptomatic from asymptomatic basilar atherosclerosis with good accuracy (Shi et al., 2018). In addition, the histogram features were also effective predictive parameters in the differences between culprit and non-culprit lesions (Shi et al., 2020). Radiomics methods show great potential in the evaluation of atherosclerotic diseases. However, to the best of our knowledge, there are no studies on the radiomics signature of plaques on VW-HRMRI in patients with SICAS.

The purpose of this study was to develop and validate a nomogram constructed by combined clinical high-risk factors, plaque radiological features, and radiomics features that can be used to predict stroke recurrence in SICAS. This nomogram could be a convenient and useful clinical tool for determining treatment strategies in patients with SICAS.

## Materials and Methods

### Patients

The data of 468 patients with SICAS who received intracranial VW-HRMRI in our hospital between September 2017 and March 2021 were extracted from the hospital database and retrospectively analyzed. Patients were eligible for inclusion (Supplementary Figure 1) if (1) they had intracranial atherosclerotic stenosis of 50–99%; (2) they had presented with ischemic stroke or symptoms of transient ischemic attack (TIA); and (3) diffusion-weighted imaging (DWI) had showed acute infarction located in the ipsilateral intracranial atherosclerotic stenosis area. The exclusion criteria were (1) ipsilateral internal carotid artery stenosis >50% on ultrasound or magnetic resonance angiography (MRA) or computed tomography angiography (CTA); (2) stroke due to non-atherosclerotic vascular disease such as dissection, vasculitis, or moyamoya disease; (3) evidence of cardiogenic embolism; or (4) poor imaging quality. All patients underwent DWI, time-of-flight (TOF)-MRA, and VW-HRMRI within 7 days of onset of symptoms, and all received standard antithrombotic therapy and statins and secondary preventive measures (control of high blood pressure and diabetes mellitus, and advice regarding smoking cessation, exercise, and weight loss).

The 156 eligible patients were divided (in a ratio of 7:3) into a training cohort (*n* = 110; 67 females, 43 males; mean age, 54 years) and a validation cohort (*n* = 46; 13 females, 33 males; mean age, 56 years).

Baseline demographic and clinical information (sex, age, hypertension, diabetes, hyperlipidemia, smoking status, stroke history, etc.) were recorded. Stroke recurrence was confirmed by a neurologist with more than 5 years of experience; for the diagnosis of recurrence, the following conditions had to be met (Coull and Rothwell, 2004): (1) sudden onset of new focal neurological defects in the previously affected region, with symptoms lasting more than 24 h and a definite imaging evidence or (2) more than two symptoms of TIA with definite distribution in the region of the intracranial atherosclerotic stenosis, even in the absence of imaging evidence of stroke, and (3) cerebral hemorrhage, tumor, and other causes ruled out.

This retrospective study was approved by the ethics committee of Shaanxi provincial people's hospital, and all patients signed the informed consent form.

### MRI Protocol

Magnetic resonance imaging was performed with a 32-channel head coil Philips 3.0T magnetic resonance scanner. The MRI sequences included T1-weighted imaging (T1WI), T2-weighted imaging (T2WI), fluid-attenuation inversion-recovery (FLAIR), and DWI. HRMRI was performed within 1 week after the onset of symptoms and included three-dimensional (3D)-TOF MRA, 3D-T1WI-volumetric isotropic TSE acquisition (VISTA), T2WI-turbo spin-echo (TSE), and 3D-T1-VISTA-enhanced imaging. The imaging parameters for each of these sequences were as follows: (1) TOF-MRA: repetition time/echo time (TR/TE) 20/3.6 ms, flip angle 18°, field of view (FOV) 180 ×180 mm^2^, matrix 256 × 256, layer thickness 0.5 mm; (2) 3D-T1WI-VISTA and 3D-T1WI-VISTA-enhanced imaging: TR/TE 700/16 ms, flip angle 90°, FOV 180 × 180 mm^2^, spatial resolution 0.5 × 0.5 × 0.5 mm^3^; and (3) T2WI-TSE: TR/TE 2,500/67 ms, FOV 80 × 80 mm^2^, matrix 256 × 256, layer thickness 2 mm. For enhanced 3D-T1WI-VISTA, gadobutrol (7.5 mL, 0.1 mmol/kg body weight) was injected intravenously and scanning was performed after 5 min; the scanning time of the whole sequence was about 30 min.

### Image Analysis

The raw VW-HRMRI data were imported into MRI-PlaqueView post-processing software (VP Diagnostics, Seattle, WA, USA), and axial, coronal, and sagittal reconstructions were generated. Two senior neuroradiologists (JG and NM, each with 5 years experience in interpreting VW-HRMRI), blinded to the clinical information, independently analyzed the images for lumen size and wall thickness. The inner lumen and outer wall were manually outlined at the maximum intraluminal narrowing (MLN) site on reconstructed post-contrast T1WI images of each patient. The reference site was the nearest plaque-free segments proximal or distal to the MLN site, and the stenosis rate, enhancement ratio, plaque burden, and remodeling index (RI) were calculated using the following formulas:

(1) The stenosis rate =1 – (Ds/Dn), where Ds was the diameter of the artery where the stenosis was most obvious and Dn was the diameter of the proximal normal artery (Samuels et al., 2000).
(2) Plaque burden = (total wall area – lumen area)/total wall area (Tian et al., 2021).
(3) The enhancement ratio was calculated at the site of maximum plaque enhancement, and the signal intensity (SI) was standardized by adjacent gray matter. The enhancement ratio of plaque was calculated using the formula: (post-contrast SI of plaque/post-contrast SI of gray matter)/(pre-contrast SI of plaque/pre-contrast SI of gray matter) (Shi et al., 2018).
(4) RI = vascular area_MLN_/vascular area_reference_. RI ≥ 1.05 was considered as positive remodeling, RI ≤ 1.05 as negative remodeling, and 0.95 < RI < 1.05 as no reconstruction (Xu et al., 2010).

### Radiomics Analysis

Radiomics extracts high-throughput quantitative image features. The work flow includes plaques segmentation, feature extraction and screening, and model building and evaluation.

### Plaque Segmentation

Three-dimensional manual segmentation was performed by a radiologist with 5 years of experience in VW-HRMRI using ITK-SNAP software (http://www.itksnap.org). The original image was magnified by four times and the regions of interest (ROIs) were drawn layer by layer on the 3D-T1WI-VISTA, T2WI-TSE, and 3D-T1WI-VISTA-enhanced images of each patient (Supplementary Figure 2). Then, the volume of the lesion was sketched along the boundary of the plaque to generate the 3D volume of interest (VOI).

### Feature Extraction

Wavelet transform was used to filter the original data and extract high-dimensional features from different frequency scales. A total of 402 imaging features reflecting plaque heterogeneity were extracted from 3D-T1WI-VISTA, T2WI-TSE, and 3D-T1WI-VISTA-enhanced images of each patient. These features included non-texture features (such as shape, size, and intensity) and texture features [gray co-occurrence matrix (GLCM), gray-level run-length matrix (GLRLM), gray-level size zone matrix (GLSZM), and neighborhood gray-tone difference matrix (NGTDM)]. All features were extracted by Matlab 2013b (The MathWorks, Natick, MA, USA). To verify the repeatability of the extraction, 20 patients in the training group were randomly selected and re-segmented by a radiologist with 10 years of experience in the interpretation of vessel wall features in MRI, and the radiomics data were measured. Intraclass correlation coefficient (ICC) was calculated to determine the repeatability.

### Clinical Features and Radiological Features

Normally distributed continuous variables were expressed as means ± standard deviation and non-normally distributed continuous variables as medians (25th−75th percentiles); comparison between the groups was performed using the independent sample *t*-test or the Mann–Whitney *U*-test, respectively. Categorical variables were summarized as counts and percentages and compared using the chi-square or Fisher exact test. Variables (clinical factors and radiological features) significantly associated (*P* < 0.1) with recurrent stroke in univariable analysis were included in multivariable analysis to identify the independent predictors of recurrent stroke.

### Identification of Radiomics Features Related to Stroke Recurrence

Least absolute shrinkage and selection operator (LASSO) were used for eigenvalue dimension reduction to select the plaque features most strongly associated with stroke recurrence in SICAS, parameter tuning conducted by 5-fold cross-validation, based on the radiomics score (Rad score) calculated for each patient with the selected feature. The Mann–Whitney *U*-test was used to analyze the relationship between radiomics features and stroke recurrence in the training and validation cohorts.

### Construction and Validation of the Nomogram

Plaque radiomics features, radiological features, and clinical risk factors were entered into a multivariable logistic regression model to identify the independent predictors of stroke recurrence in the training cohort. The identified predictors were then used to construct the radiomics nomogram for quantifying the risk of stroke recurrence in the training cohort. The efficacy of the nomogram was tested on the internal validation cohort; the total score of each patient was calculated and the association with stroke recurrence was examined. Finally, the C-index and calibration curve were generated from the regression model. To evaluate the clinical effectiveness of the nomogram, decision curve analysis (DCA) was used to quantify the net benefit probability under different thresholds in the training and verification cohorts.

### Evaluation of the Predictive Nomogram

Receiver operating characteristic (ROC) analysis was used to assess the usefulness of the nomogram. The Delong test and Bonferroni correction were used to correct *p*-values for multiple comparisons. The AUC, with 95% confidence intervals (CI), sensitivity, specificity, and accuracy were calculated.

### Statistical Analysis

Data analysis was done with SPSS 22 (IBM Corp., Armonk, NY, USA) and R 3.6.3 (https://cran.r-project.org/bin/windows/base/old/3.6.3/). *P* ≤ 0.05 indicated statistically significant difference.

## Results

### Comparison of Clinical and Radiological Features

Among the 156 patients in this study, the middle cerebral artery was involved in 97 patients and the basilar artery in 59 patients. Recurrence occurred in 32/110 (29.1%) patients in the training cohort and 13/46 (28.3%) in the validation cohort. Table 1 presents a comparison of clinical factors and radiological features between the training cohort and the validation cohort. Both continuous (plaque burden, plaque enhancement ratio, plaque thickness, and remodeling index) and categorical (diabetes mellitus) variables were associated with stroke recurrence in univariable analysis. In multivariable logistic regression analysis, the independent predictors of stroke recurrence were diabetes mellitus [odds ratio (OR) = 1.24, 95% CI: 1.04–3.79, *p* = 0.018], plaque burden (OR = 1.76, per 10% increase, 95% CI: 1.28–2.41, *p* < 0.001), and plaque enhancement ratio (OR = 1.94, 95% CI: 1.27–3.09, *P* < 0.001).

**Table 1:** Clinical and radiological features of the SICAS in the training and validation cohort.

**Characteristics**	**Training (*****n*** **= 110)**			**Validation (*****n*** **= 46)**		
	**Recurrence (*n* = 32)**	**No-recurrence (*n* = 78)**	***P*-value**	**Recurrence (*n* = 13)**	**No-recurrence (*n* = 33)**	***P*-value**
Sex, males	12 (37.5)	31 (39.7)	0.827	8 (61.5)	25 (75.8)	0.335
Age, y	53.3 ± 14.6	56.5 ± 14.4	0.293	57.15 ± 8.57	54.09 ± 13.45	0.451
Diabetes mellitus	20 (62.5)	27 (34.6)	0.007	10 (76.9)	9 (27.2)	0.003
Hypertension	14 (43.8)	42 (53.8)	0.336	4 (30.8)	20 (60.6)	0.103
Current smoker	18 (56.3)	51 (65.4)	0.368	8 (61.5)	19 (57.6)	0.806
Hyperlipidemia	14 (43.8)	23 (29.5)	0.15	6 (46.2)	9 (27.3)	0.219
Stroke history	10 (31.3)	32 (41)	0.604	6 (46.2)	8 (24.2)	0.146
Plaque burden	0.70 ± 0.14	0.64 ± 0.09	0.007	0.76 ± 0.11	0.63 ± 0.12	0.001
Plaque thickness	1.81 ± 0.83	1.34 ± 0.73	0.13	1.93 ± 0.72	1.37 ± 0.84	0.04
Enhancement ratio	2.7 ± 1.03	2.12 ± 0.94	0.002	2.75 ± 1.19	2.07 ± 1.0	0.01
Stenosis, %	0.75 ± 0.14	0.76 ± 0.13	0.948	0.74 ± 0.16	0.75 ± 0.11	0.766
Positive remodeling	25 (78.1)	45 (57.7)	0.043	10 (76.9)	17 (51.5)	0.184

*Data are presented as mean ± standard deviation or n (%)*.

### Radiomics Signature of Plaque for Predicting Stroke Recurrence

**Figure 1:**
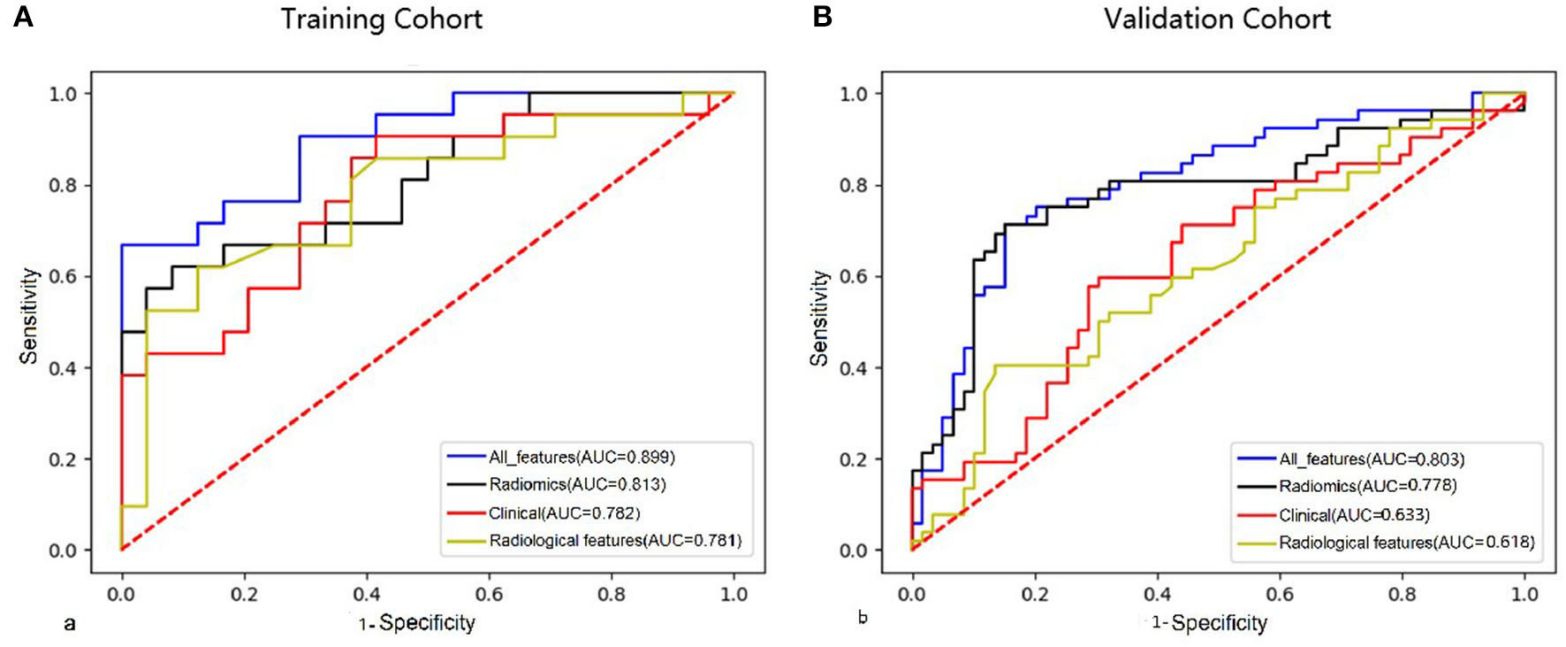
Comparison of ROC curves of clinical features, radiological features, combined radiomics features, and all features in the training cohort **(A)** and validation cohort **(B)**.

The LASSO regression model was used to extract the MRI features with the most predictive value for stroke recurrence in the training cohort; they included six features in 3D-T1WI-VISTA, nine features in T2WI, and seven features in 3D-T1WI-VISTA-enhanced imaging (Table 2). There were significant differences in rad scores for different sequences between patients with and without recurrent stroke in the training cohort: 3D-T1WI-VISTA: −0.43 ± 0.76 vs. 0.14 ± 0.71 (*p* < 0.001); T2WI: −0.46 ± 0.41 vs. −0.23 ± 0.37 (*p* < 0.002); 3D-T1WI-VISTA-enhanced imaging: −0.71 ± 0.73 vs. −0.24 ± 0.63 (*p* < 0.001). Among the individual sequences, the radiomics signature on 3D-T1WI-VISTA-enhanced imaging was superior to the others for predicting stroke recurrence in both the training cohort (AUC, 0.790, 95% CI: 0.669–0.894) and the validation cohort (AUC, 0.779, 95% CI: 0.620–0.853). The combination of all three sequences (3D-T1WI-VISTA, T2WI, and 3D-T1WI-VISTA-enhanced) had the best predictive value in both the training cohort (AUC, 0.813, 95% CI: 0.741–0.901) and the validation cohort (AUC, 0.778, 95% CI: 0.690–0.878) (Table 3 and Figure 1).

**Table 2:** Effective intercept features for the 3DT1WI-VISTA, T2WI, and 3DT1WI-VISTA-enhanced images in SICAS.

	**Coefficients**	**Intercept feature**
3DT1WI-VISTA (*n* = 6)	0.69	GLCM Entropy_AllDirection_offset7
	−0.22	Mean Deviation
	0.62	Histogram Energy
	1.32	Maximum3DDiameter
	0.09	Cluster Shade_angle0_offset7
	0.16	GLCMEntropy_AllDirection_offset1_SD
T2WI (*n* = 9)	−0.25	Relative Deviation
	0.50	GLCMEntropy_AllDirection_offset1_SD
	−0.24	GLCMEnergy_angle90_offset7
	−0.17	GreyLevelNonuniformity_AllDirection_offset4_SD
	0.03	GreyLevelNonuniformity_AllDirection_offset1_SD
	−0.32	GLCMEnergy_angle135_offset7
	−0.37	GLCMEnergy_angle0_offset7
	1.21	Maximum3DDiameter
	0.34	GLCMEntropy_AllDirection_offset4
3DT1WI-VistaCE (*n* = 7)	−0.34	Small Area Emphasis
	−0.14	GLCMEntropy_angle135_offset7
	−0.15	ShortRunHighGreyLevelEmphasis_AllDirection_offset7_SD
	−0.54	ShortRunEmphasis_angle90_offset1
	0.94	Maximum3DDiameter
	−1.80	GLCMEntropy_angle90_offset4
	−0.21	Correlation_AllDirection_offset7_SD

**Table 3:** Prediction efficiency of different models in the training and validation cohorts.

**Model**	**Training cohort (*****n*** **= 110)**				**Validation cohort (*****n*** **= 46)**			
	**Accuracy**	**Sensitivity**	**Specificity**	**AUC (95% CI)**	**Accuracy**	**Sensitivity**	**Specificity**	**AUC (95% CI)**
Clinical features	0.667	0.571	0.750	0.782 (95% CI: 0.563–0.860)	0.631	0.538	0.712	0.633 (95% CI: 0.518–0.802)
Radiological features	0.756	0.624	0.958	0.781 (95% CI: 0.685–0.904)	0.641	0.584	0.864	0.618 (95% CI: 0.517–0.857)
Clinical + Radiological features	0.773	0.763	0.705	0.801 (95% CI: 0.633–0.891)	0.659	0.717	0.622	0.726 (95% CI: 0.604–0.879)
3D-T1WI-VISTA Radiomics signature	0.667	0.619	0.708	0.744 (95% CI: 0.603–0.865)	0.703	0.731	0.678	0.737 (95% CI: 0.586–0.855)
T2WI Radiomics signature	0.615	0.746	0.846	0.750 (95% CI: 0.596–0.917)	0.733	0.834	0.619	0.717 (95% CI: 0.566–0.836)
3D-T1WI-VISTA-enhanced Radiomics signature	0.748	0.757	0.826	0.790 (95% CI: 0.669–0.894)	0.711	0.667	0.75	0.779 (95% CI: 0.620–0.853)
Combined radiomics signature	0.756	0.767	0.833	0.813 (95% CI: 0.741–0.901)	0.776	0.712	0.831	0.778 (95% CI: 0.690–0.878)
All features	0.822	0.844	0.917	0.899 (95% CI: 0.844–0.936)	0.757	0.814	0.847	0.803 (95% CI: 0.761–0.897)

### Predictive Model for Stroke Recurrence

The nomogram for prediction of recurrent stroke included clinical risk factors, radiological features, and radiomics signature (Figure 1 and Supplementary Figure 3). In the training cohort, the AUC of the nomogram was 0.899 (95% CI: 0.844–0.936), the sensitivity was 0.844, the specificity was 0.917, and the accuracy was 0.822 (Table 3). In the validation cohort, the AUC was 0.803 (95% CI: 0.761–0.897), the sensitivity was 0.814, the specificity was 0.847, and the accuracy was 0.757 (Table 3). Moreover, the AUC of the nomogram with the training cohort and the validation cohort were better than clinical features (0.899 vs. 0.782, *p* = 0.003; 0.803 vs. 0.633; *p* = 0.002) and radiological features (0.899 vs. 0.781; *p* = 0.003; 0.803 vs. 0.618; *p* =0.001). However, the AUC of the nomogram model was superior to radiomics features in the training cohort (0.899 vs. 0.813; *p* = 0.021) and did not differ from the validation cohort (0.803 vs. 0.778; *p* = 0.626).

### Development and Validation of the Nomogram

**Figure 2:**
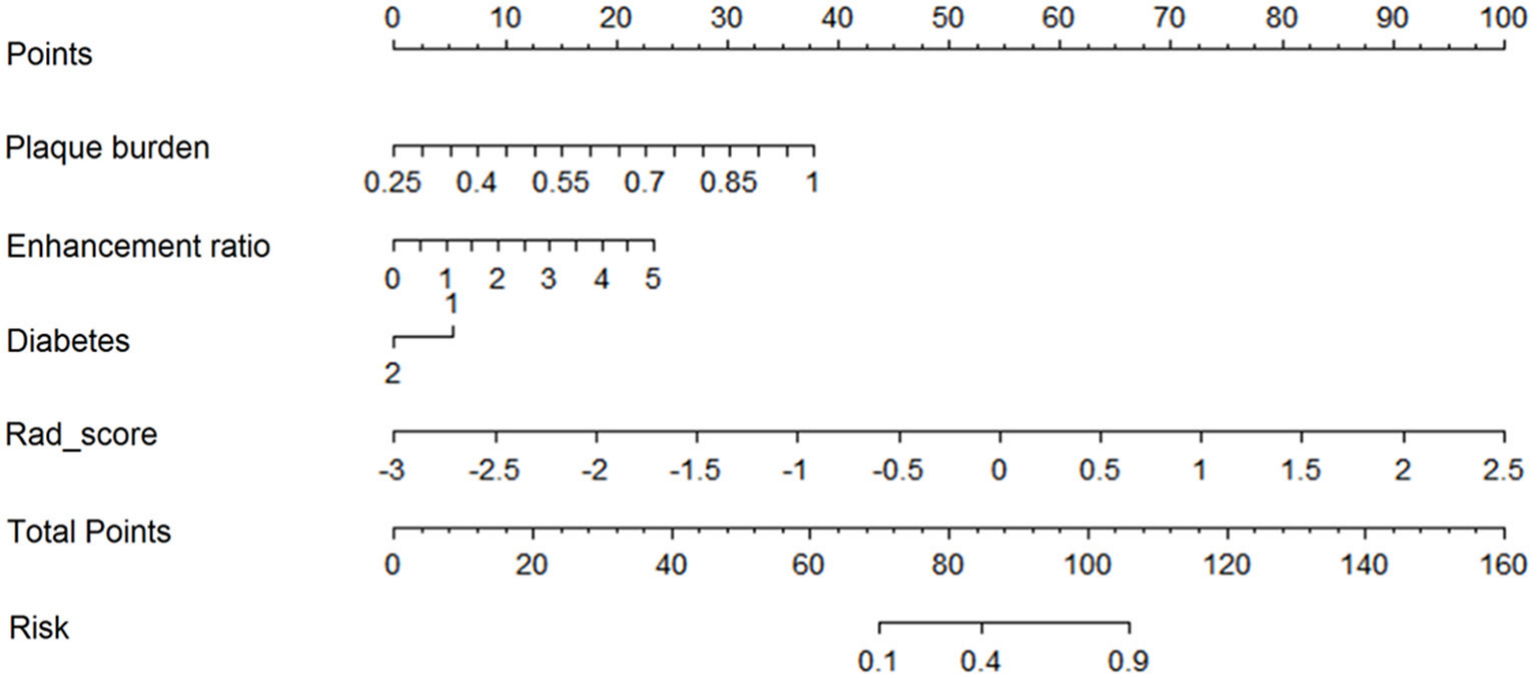
Nomogram for prediction of stroke recurrence in SICAS.

**Figure 3:**
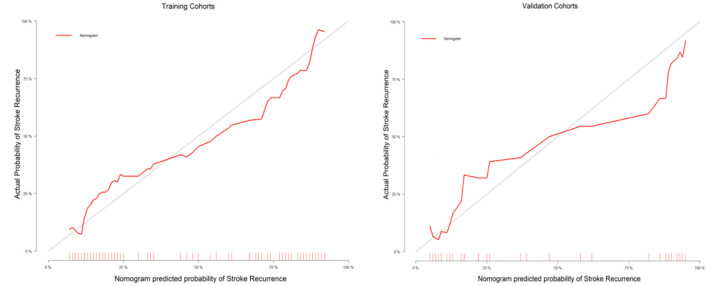
Calibration curves of the nomogram in the training and validation cohorts.

**Figure 4:**
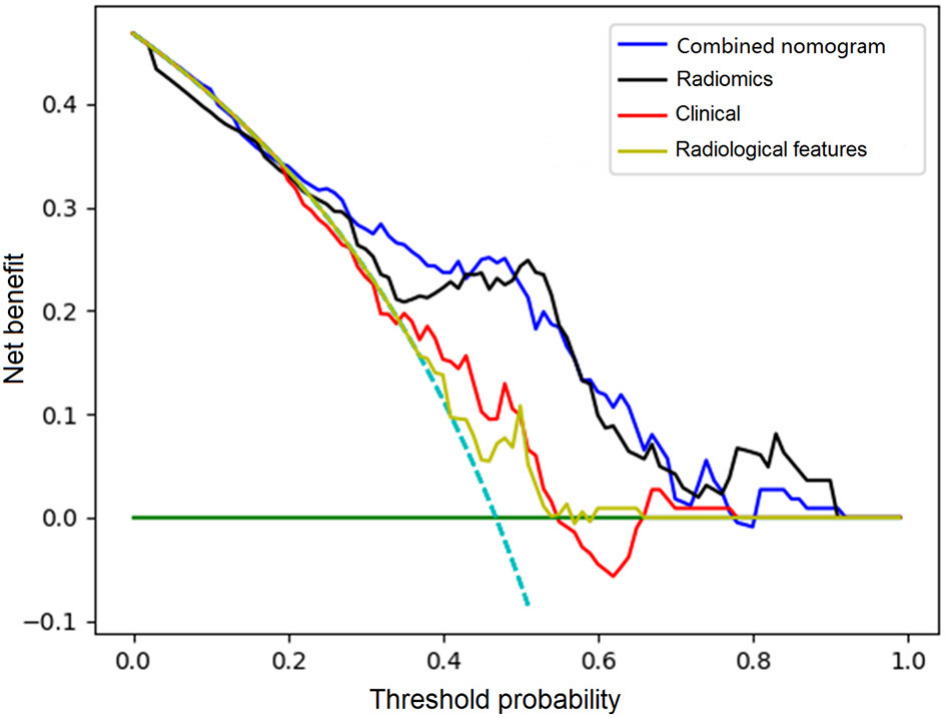
Comparison of decision curves analysis for clinical features, radiological features, combined radiomics features, and the constructed nomogram for prediction of stroke recurrence in SICAS.

The C-index of the nomogram was 0.880 (95% CI: 0.805–0.934) and 0.817 (95% CI: 0.795–0.948) in the training cohort and validation cohort, respectively, indicating good accuracy in predicting stroke recurrence in SICAS (Figure 2). The calibration curve showed that the nomogram-predicted probability was close to the actual probability of stroke in the training cohort and validation cohort (*p* = 0.901 and *p* = 0.548, respectively; Figure 3). The threshold probability of the decision curve was 2% and the net profit rate was 0.458. When the threshold probability of decision curve was >22%, the nomogram-predicted probability of stroke recurrence was higher than the probability predicted by clinical risk factors or radiological features alone (Figure 4).

### Interobserver Consistency

There was good interobserver agreement in radiomics signature extraction and quantitative feature of plaque image (ICC, 0.761–0.898).

## Discussion

This study showed that diabetes mellitus, plaque burden, and plaque enhancement ratio are independent predictors of stroke recurrence in SICAS; moreover, radiomics features of plaque on HRMRI had a high sensitivity for identifying the risk of recurrent stroke. The predictive nomogram constructed using high-risk clinical factors, radiological features, and radiomics features of plaques showed a high accuracy for the personalized prediction of stroke recurrence. These findings were helpful for early clinical intervention in people with a high risk of stroke recurrence.

Plaque enhancement ratio and plaque burden have been previously shown to be significantly associated with stroke recurrence in SICAS (Ran et al., 2020; Shi et al., 2020; Xiao et al., 2022). Plaque enhancement, a characteristic of vulnerable plaque, is related to plaque inflammation, neovascularization, and vascular endothelial permeability (Moulton et al., 2003; Marnane et al., 2012; Millon et al., 2012; Qiao et al., 2014). Our study confirmed that both plaque enhancement ratio and plaque burden, rather than degree of stenosis, are major predictors of stroke recurrence in SICAS. Several studies from coronary/intracranial artery disease have suggested the limitation of intraluminal imaging in predicting future vascular events, and the presence of stenosis may not significantly increase the risk of future vascular events (Kim et al., 2016; Mortensen et al., 2020; Ran et al., 2020). In a word, it is not enough to use the degree of stenosis to identify real high-risk patients with stroke recurrence in SICAS who may benefit from preventive treatment. The radiomics features of plaque were better than the conventional HRMRI plaque image characteristics for predicting recurrent stroke in SICAS. Among all HRMRI sequences, 3D-T1WI-VISTA-enhanced images had the highest predictive value. The combination of 3D-T1WI-VISTA, T2WI, and 3D-T1WI-VISTA-enhanced images had an excellent value for predicting stroke recurrence in the training and validation cohorts. Thus, the present study showed that radiomics can excavate quantitative texture and non-texture features that cannot be distinguished with the naked eye.

In our study, 11 radiomics signatures were associated with recurrent stroke in SICAS when combining constructing radiomics signatures. Stroke recurrence in atherosclerotic stenosis has been shown to be closely related to the morphological characteristics of plaque (Roquer et al., 2011; Altaf et al., 2014; Deng et al., 2020). This is consistent with our study, which found plaque thickness (in the validation cohort) and positive plaque remodeling (in the training cohort) to be more in the recurrence group than in the non-recurrence group. The finding that Maximum_3D_Diameter is based on morphology radiomics feature, which is related to SICAS recurrence, supports this deduction. Combined with the radiomics model, Histogram Energy represents the degree of gray-scale dispersion, which is related to stroke recurrence in SICAS; it may indicate that the heterogeneity of plaques (lipid-rich necrotic core, inflammation, and hemorrhage) in the SICAS recurrence group is more significant than the non-recurrence group (Salem et al., 2012). A recent study has also demonstrated that the coefficient of variation of histogram analysis was capable of differentiating culprit and non-culprit lesions for ICAS (Shi et al., 2021). Compared with non-culprit lesions, culprit lesions had a larger coefficient of variation as a consequence of a larger difference between the minimum and maximum intensity value and/or smaller mean intensity value on T1WI. The advantage of the radiomics method is that it can provide more information than conventional imaging (Gillies et al., 2016). In the present study, stroke recurrence in SICAS was associated with nine other texture features; these radiomics signatures cannot be easily identified and understood by an observer, and it is still a big challenge to explain the relationship between these radiomics signatures and pathology (Tran et al., 2012), which raises the researchers' attention to the radiomics method in plaques.

In the prediction model of stroke recurrence, we included clinical risk factors, radiological features, and radiomics signature. The radiomics predictive model is better than clinical and radiological features in the prediction of stroke recurrence. The use of combined radiomics features can improve the prediction efficiency of stroke recurrence in SICAS. In addition, the nomogram has higher prediction performance and good calibration in the training cohort (C-index 0.880) and the validation cohort (C-index 0.817). Therefore, the nomogram is helpful for clinicians to provide decision-making basis for individualized treatment of stroke recurrence in SICAS.

Diabetes mellitus, along with other biomarkers, could be used to predict the risk of stroke recurrence in SICAS. Our results are consistent with those of previous studies and suggest that diabetes may be an independent risk factor for stroke recurrence (Shou et al., 2015). Therefore, to avoid the serious consequences of stroke recurrence, diabetes mellitus as a risk factor should be considered for the treatment and recovery of stroke patients.

This study has some limitations. First, the use of VOI segmentation in this study may have led to subjective errors and affected the accuracy of radiomics analysis. Second, this was a single-center retrospective study with a relatively small sample size, so selection bias cannot be ruled out.

In conclusion, radiomics features are potential biomarkers for predicting stroke recurrence in SICAS. The nomogram constructed by combining clinical high-risk factors, plaque radiological features, and radiomics features is helpful to the individualized risk assessment of predicting the stroke recurrence of SICAS.

## Data Availability Statement

The original contributions presented in the study are included in the article/Supplementary Material, further inquiries can be directed to the corresponding author/s.

## Ethics Statement

The studies involving human participants were reviewed and approved by the Ethics Committee of Shaanxi Provincial People's Hospital. The patients/participants provided their written informed consent to participate in this study.

## Author Contributions

MT, JG, and NM drafted the manuscript and designed the experiment. XY and ZZ performed the statistical analysis. XinZ, JH, XS, XL, and LL collected the data. XL provided technical support. XiaZ contributed to the design of the experiment and also revised the manuscript. All authors contributed to the article and approved the submitted version.

## Funding

This research was supported by the Shanxi Provincial Key Research and Development Project of Shaanxi Province of China (2021SF-064).

## Conflict of Interest

The authors declare that the research was conducted in the absence of any commercial or financial relationships that could be construed as a potential conflict of interest.

## Publisher's Note

All claims expressed in this article are solely those of the authors and do not necessarily represent those of their affiliated organizations, or those of the publisher, the editors and the reviewers. Any product that may be evaluated in this article, or claim that may be made by its manufacturer, is not guaranteed or endorsed by the publisher.

## Supplementary Material

Supplementary Figure 1 Flow chart of the study eligible patients.

Supplementary Figure 2 Plaque outline presentation. T2WI shows MCA atherosclerotic plaque of M1 segment of right middle cerebral artery **(A)**. Plaque were drawn layer by layer on the T2WI-TSE images **(B)**. The volume of the lesion was sketched along the boundary of the plaque to generate the 3D volume of interest (VOI) **(C)**.

Supplementary Figure 3 An example of using the nomogram to illustrate the individual risk of stroke recurrence in SICAS. A 38-years-old male was showed paroxysmal dysphasia with right limb weakness for 1 week. MRA demonstrates stenosis and plaque of M1 segment of left middle cerebral artery, the plaque burden was 0.83 (**A,B**, yellow line). The enhancement ratio of plaque was 3.3 (**C,D**, blue line). No diabetes history (purple line), Rad_score (green line). The values on the Points scale intersected by the lines were added to obtain total points (28 + 16 + 0 + 62 = 106). The total points >82 points, considered as a high-risk patient. The graph revealed that the risk of stroke recurrence in SICAS was over 82% by drawing a vertical line on the Total points scale. Cerebral infarction recurred in the distribution of the left middle cerebral artery after followed up for 6 months.

## Abbreviations

ICAS: intracranial atherosclerotic stenosis
VW-HRMRI: vessel wall high-resolution magnetic resonance imaging
HRMRI: high-resolution magnetic resonance imaging
SICAS: symptomatic intracranial atherosclerotic stenosis
AUC: area under the curve
TIA: transient ischemic attack
DWI: diffusion-weighted imaging
MRA: magnetic resonance angiography
CTA: computed tomography angiography
TOF: time-of-flight
T1WI: T1-weighted imaging
T2WI: T2-weighted imaging
FLAIR: fluid-attenuation inversion-recovery
3D: three-dimensional
TSE: turbo spin-echo
VISTA: volumetric isotropic T2W acquisition
FOV: field of view
TR/TE: repetition time/echo time
WASID: warfarin-Aspirin Symptomatic Intracranial Disease
SI: signal intensity
RI: remodeling index
VOI: volume of interest
ROI: region of interest
GLCM: gray co-occurrence matrix
GLRLM: gray-level run-length matrix
GLSZM: gray-level size zone matrix
NGTDM: neighborhood gray-tone difference matrix
ICC: intraclass correlation coefficient
LASSO: least absolute shrinkage and selection operator
Rad score: radiomics score
DCA: decision curve analysis
ROC: receiver operating characteristic
CI: confidence interval
OR: odds ratio.
