# Supplementary figures and images for: Radiomics Nomogram for Predicting Stroke Recurrence in Symptomatic Intracranial Atherosclerotic Stenosis
Source: Front Neurosci. 2022 Apr 12;16:851353. doi: 10.3389/fnins.2022.851353 (PMC9039339; doi:10.3389/fnins.2022.851353)

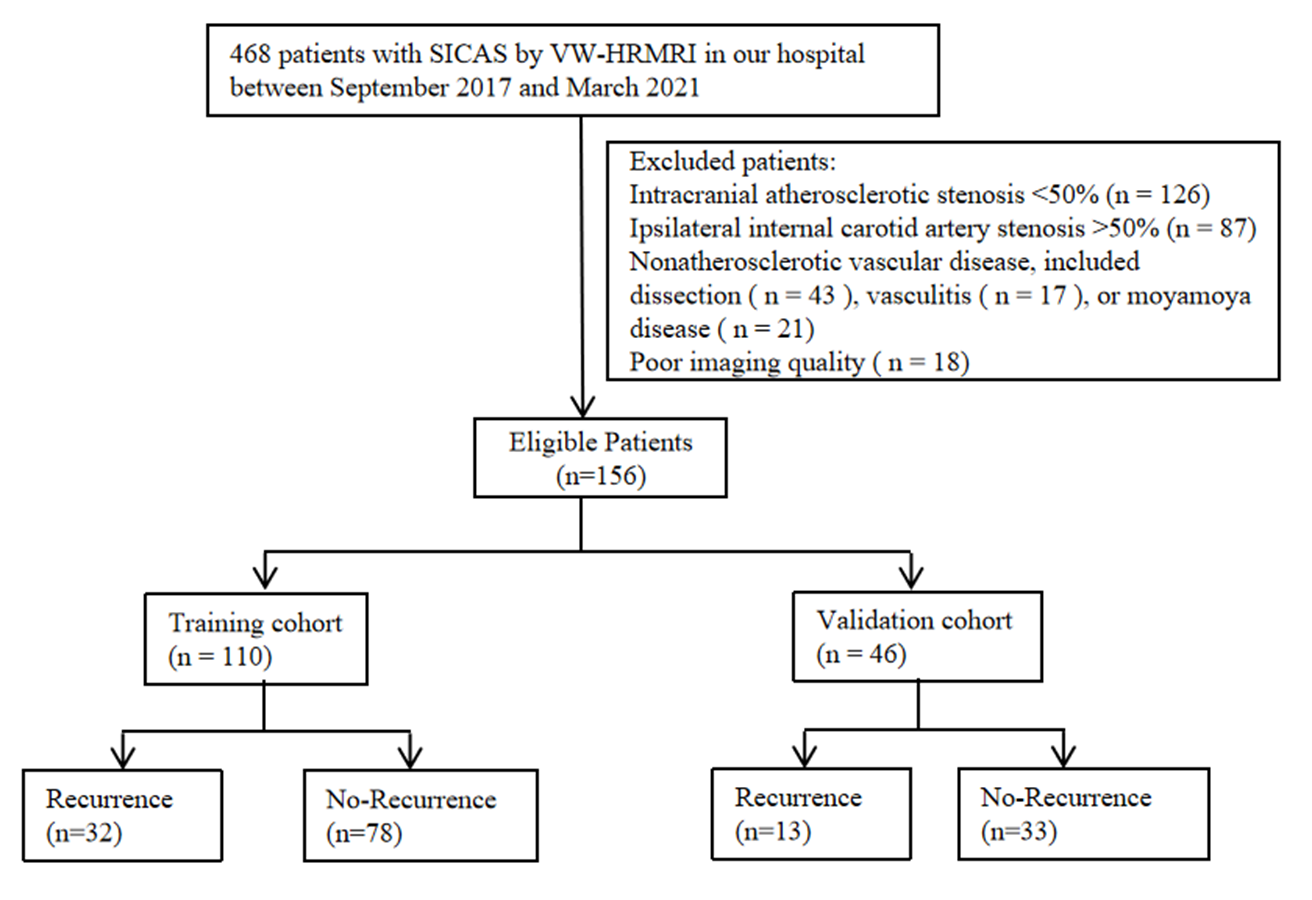

Supplement: Supplementary Figure 1 — Flow chart of the study eligible patients. [file Image_1.TIF]

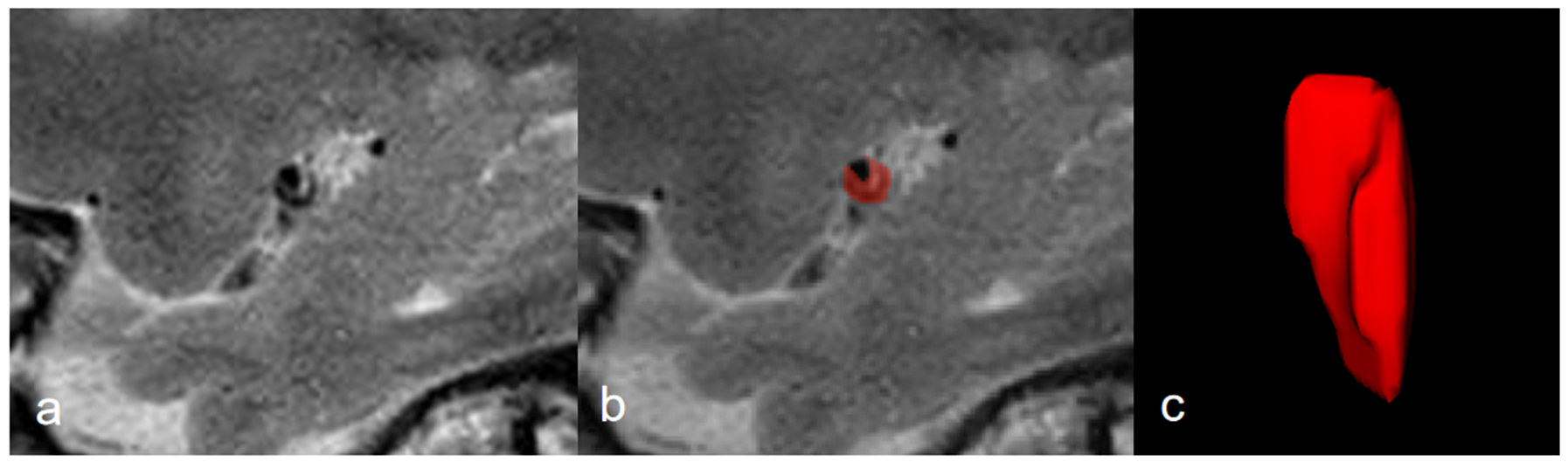

Supplement: Supplementary Figure 2 — Plaque outline presentation. T2WI shows MCA atherosclerotic plaque of M1 segment of right middle cerebral artery (A). Plaque were drawn layer by layer on the T2WI-TSE images (B). The volume of the lesion was sketched along the boundary of the plaque to generate the 3D volume of interest (VOI) (C). [file Image_2.TIF]

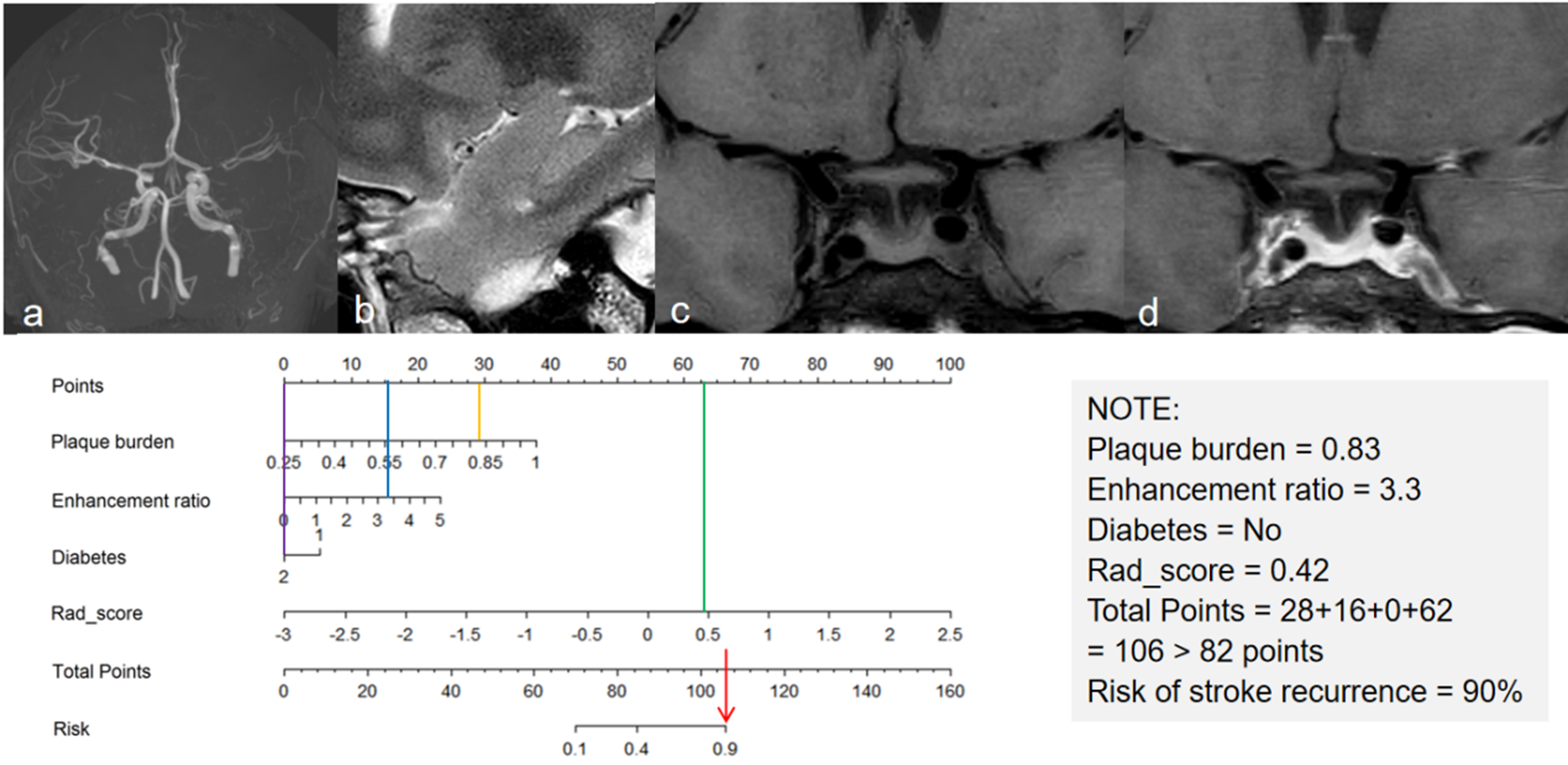

Supplement: Supplementary Figure 3 — An example of using the nomogram to illustrate the individual risk of stroke recurrence in SICAS. A 38-years-old male was showed paroxysmal dysphasia with right limb weakness for 1 week. MRA demonstrates stenosis and plaque of M1 segment of left middle cerebral artery, the plaque burden was 0.83 (A,B, yellow line). The enhancement ratio of plaque was 3.3 (C,D, blue line). No diabetes history (purple line), Rad_score (green line). The values on the Points scale intersected by the lines were added to obtain total points (28 + 16 + 0 + 62 = 106). The total points >82 points, considered as a high-risk patient. The graph revealed that the risk of stroke recurrence in SICAS was over 82% by drawing a vertical line on the Total points scale. Cerebral infarction recurred in the distribution of the left middle cerebral artery after followed up for 6 months. [file Image_3.TIF]
